# Structural biology of human telomerase: progress and prospects

**DOI:** 10.1042/BST20200042

**Published:** 2021-10-08

**Authors:** Thi Hoang Duong Nguyen

**Affiliations:** Structural Studies Division, Medical Research Council Laboratory of Molecular Biology, Cambridge, U.K.

**Keywords:** cryo-EM, human telomerase, telomeres

## Abstract

Telomerase ribonucleoprotein was discovered over three decades ago as a specialized reverse transcriptase that adds telomeric repeats to the ends of linear eukaryotic chromosomes. Telomerase plays key roles in maintaining genome stability; and its dysfunction and misregulation have been linked to different types of cancers and a spectrum of human genetic disorders. Over the years, a wealth of genetic and biochemical studies of human telomerase have illuminated its numerous fascinating features. Yet, structural studies of human telomerase have lagged behind due to various challenges. Recent technical developments in cryo-electron microscopy have allowed for the first detailed visualization of the human telomerase holoenzyme, revealing unprecedented insights into its active site and assembly. This review summarizes the cumulative work leading to the recent structural advances, as well as highlights how the future structural work will further advance our understanding of this enzyme.

## Progress

### Introduction

In the 1930s, the natural ends of chromosomes were independently discovered in maize and fruit flies by McClintock and Muller, respectively [1–3]. Unlike DNA breaks, these ends were shown to have a special ability to escape chromosome end-to-end fusions, and subsequently named telomeres [3]. After the discovery of DNA structure [4–6], the mechanisms governing DNA replication emerged, and the end-replication problem was realized: linear chromosome ends are incompletely copied by the replication machinery. This results in a gradual sequence loss at the telomeres [7,8]. However, it was unclear how cells solved this problem. In the late 1970s, Blackburn and colleagues found that telomeric DNA from the ciliate *Tetrahymena thermophila* consisted of repetitive TTGGGG sequences [9]. The ‘terminal transferase’ responsible for synthesizing this sequence was subsequently discovered in *Tetrahymena* cell extract by Greider and Blackburn [10] and named *telomerase*.

Human telomerase activity was later detected in HeLa cells [11]. Further analyses of other human cell lines and tissues revealed that this activity was undetectable in normal somatic cells, but present in immortal cell lines, such as cancer cells, stem cells and germline cells [12,13]. These findings link telomerase expression to cell immortalization. Intuitively, telomerase expression allows these cells to maintain stable telomere lengths, which would otherwise shorten due to the end-replication problem [13,14].

From decades of research on telomeres and telomerase, we now know that, like *Tetrahymena*, telomeres of most eukaryotic cells consist of tandem arrays of repetitive G-rich sequences (TTAGGG in mammals) with a 3′ overhang on the G-strand. Although telomeres play key roles in protecting the chromosomes from end-resection and inter-chromosome fusion [15], they are progressively shortened due to the end-replication problem [14]. To compensate for this telomere loss, telomeric DNA is specifically lengthened by telomerase [16].

### Telomerase — a specialized reverse transcriptase

Compared with the commonly studied retroviral reverse transcriptases, telomerase is unique in at least two major ways [16,17]. First, *de novo* synthesis of telomeric repeats at chromosome ends by telomerase requires both the reverse transcriptase activity of telomerase reverse transcriptase (TERT) subunit and an internal RNA template embedded within telomerase RNA (TER or hTR in humans). Second, unlike retroviral reverse transcriptases, telomerase can add multiple telomeric repeats to a single DNA substrate before dissociation — a property called repeat addition processivity. These special features led to numerous phylogenetic, genetic, biochemical and structural studies to determine how TERT and TER co-ordinate telomerase DNA synthesis and how repeat addition processivity is achieved [17].

**Figure 1.**
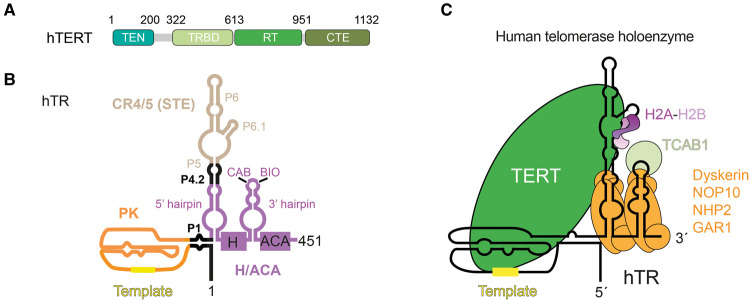
Schematic presentation of human telomerase. (**A**) Domain architecture of human telomerase reverse transcriptase (hTERT). TEN, telomerase essential N-terminal domain; TRBD, telomerase RNA binding domain; RT, reverse transcriptase; CTE, C-terminal extension. (**B**) Secondary structure presentation of human telomerase RNA (hTR). PK, pseudoknot; CR4/5, conserved regions 4 and 5; H/ACA, box H and box ACA; STE, stem-terminus element. P5, P6 and P6.1 of the CR4/5 and the 5′ and 3′ RNA hairpins of the H/ACA domain are labelled. (**C**) Schematic of human telomerase holoenzyme.

Across different eukaryotes, TERT shares four conserved domains: telomerase essential N-terminal (TEN) domain, telomerase RNA binding domain (TRBD), reverse transcriptase (RT) domain and C-terminal extension (CTE) domain [18,19] (Figure 1A). In contrast, TERs are highly divergent in size, primary sequence and secondary and likely tertiary structure. TERs also have different requirements for biogenesis [20]. Phylogenetic analyses show that all TERs contain two conserved structural elements essential for telomerase catalytic activity: a pseudoknot–template (PK/t) domain and stem-terminus element (STE) [21–24]. The PK/t domain, the most conserved feature of TERs, consists of the template region for telomeric DNA synthesis and an adjacent pseudoknot fold at the 3′ end of the template (Figure 1B). On the other hand, the STE can exist as a single stem–loop, as found in ciliates and flagellates, or as a three-way junction, as found in fungi and vertebrates [25–29]. In vertebrates, the STE is also known as conserved regions 4 and 5 [30] (Figure 1B). These structural elements associate with the domains of TERT to reconstitute telomerase activity *in vitro* [22,31] (Figure 1C).

Beyond TERT and TER, cellular telomerase holoenzymes are more complex in composition and require additional accessory factors, each of which can play essential roles for the biogenesis, localization and regulation of telomerase ribonucleoprotein in the cells [32–34]. Subunit composition requirements vary considerably across different eukaryotes, and are still the subject of ongoing research. For the scope of this review, I will focus on advances on the human telomerase holoenzyme. Henceforth, human TERT and TER will be referred to as hTERT and hTR, respectively.

### The composition of human telomerase

Although hTERT and hTR are sufficient to reconstitute a minimal telomerase catalytic core in rabbit reticulocyte lysates [31], the endogenous human telomerase complex is considerably larger and has an estimated molecular mass of 550–650 kilodaltons (kDa) [35–37]. This size estimate significantly exceeds the combined molecular mass of hTERT and hTR (289 kDa) and raised a question as to what else made up for this molecular mass difference.

The first clues came when numerous dyskeratosis congenita disease mutations, which result in compromised telomerase function, were first identified in a protein named dyskerin [38]. Dyskerin is a pseudouridine synthase known to associate with box H/ACA small nucleolar RNAs (snoRNA) at the time [39]. Curiously, the 3′ domain of hTR showed resemblance to the snoRNAs with the characteristic double RNA hairpin structure, a conserved H box between the two hairpins and an ACA box at the 3′ end [40] (Figure 1B). These lines of evidence hinted at dyskerin association with human telomerase [41]. Mass spectrometry of purified endogenous human telomerase confirmed the presence of dyskerin, leading to a proposal that human telomerase was a dimer of hTERT, hTR and dyskerin [36].

In addition to dyskerin, each RNA hairpin within the H/ACA RNA also binds three other protein subunits, namely NOP10, NHP2 and GAR1 [42] (Figure 1C). Association of dyskerin with NOP10 and NHP2 is required for dyskerin interaction with RNA [43]. Indeed, these proteins were detected by mass spectrometry of affinity-purified human telomerase holoenzyme from HeLa cells in another study [44]. Further work demonstrated that the H/ACA motif was important for the accumulation of human telomerase RNP *in vivo* and that human telomerase ribonucleoprotein assembles two copies of the H/ACA heterotetramer (dyskerin, NOP10, NHP2 and GAR1) [45]. The 3′ RNA hairpin of the hTR H/ACA domain also possesses a motif within its terminal stem loop named CAB box, which binds the Cajal body localization factor, TCAB1 (Figure 1B,C) [46,47]. From these findings, the monomeric hTERT/hTR model of human telomerase holoenzyme was proposed. In this model, telomerase is composed of hTERT, hTR, two sets of the H/ACA heterotetramer and TCAB1. Both this model and the hTERT/hTR/dyskerin dimer model yield molecular mass close to the initial estimates. However, as I will describe next, subsequent structural studies would provide the necessary resolution to revise the functional compositional model of human telomerase.

### An overview of electron microscopy studies of human telomerase

As a ribonucleoprotein, telomerase requires a complex biogenesis/assembly pathway [32]. This makes it challenging to produce the sample recombinantly from purified constituents, especially when the composition was still unclear. Furthermore, the scarcity of the endogenous complex [36,48] poses a significant challenge for sample preparation from an endogenous source in the quantity and quality needed for structural studies. Thus, high-resolution X-ray or NMR structural studies of telomerase had been limited to truncated TERT and domains of TER from both humans and other species [49,50]. These two techniques require milligram quantities, which has yet to be achieved with the entire human telomerase. Additionally, the complexity and inherent flexibility of a such multi-subunit assembly would preclude it from forming well-ordered crystals. Both the low quantity and flexibility issues could be overcome with cryo-electron microscopy (cryo-EM). Furthermore, recent technological developments have allowed structures of challenging biological macromolecules to be determined by cryo-EM at an atomic resolution [51,52], presenting an exciting opportunity for the telomerase field.

A breakthrough in sample preparation arose from the development of an overexpression system by transient transfection of human cells with hTR and hTERT [53,54]. Overexpressed hTERT and hTR are assembled with other more abundant holoenzyme factors via an endogenous assembly pathway. The yield of telomerase obtained is substantially higher when compared with the endogenous levels. Affinity tags and mutations can also be introduced for purification and biochemical characterization of the complex [55–57]. These methods have been used extensively in many studies across the field, including the structural work described next.

**Figure 2.**
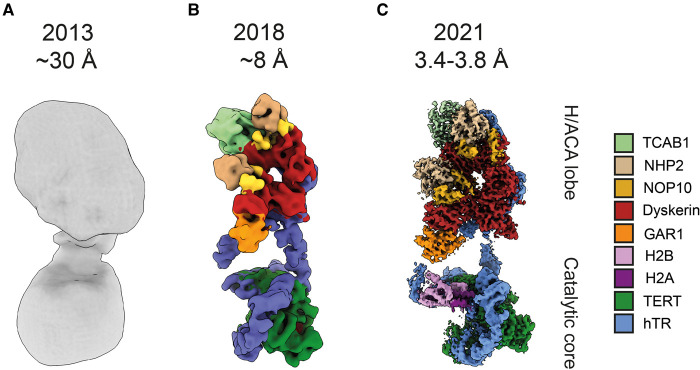
Structures of human telomerase holoenzyme determined by electron microscopy. (**A**) 30 Å negative stain EM structure. (**B**) 8 Å cryo-EM structure. (**C**) Sub-4 Å cryo-EM structure. For (**B**,**C**), composite maps of the two lobes of the structures are shown.

The first glimpse of human telomerase was provided by the 30 Å negative stain EM structure of the complex (Figure 2A) [56]. The sample was obtained using tandem affinity purification using tags on hTERT coupled with ion-exchange chromatography and GraFix [58]. The structure displayed a bilobed architecture with a flexible linker connecting the lobes (Figure 2A). To address whether this structure consisted of a telomerase monomer or dimer, hTERT molecules were indirectly counted with gold-labelled telomeric DNA. The experiments indicated the existence of telomerase particles without gold, one gold and two gold particles bound; and the population with one gold particle was the most abundant. It was reasoned that dimeric telomerase could bind up to two gold particles, and that incomplete DNA binding resulted in telomerase molecules without gold or with only one gold particles. Together with additional experiments involving differential tagging of hTERT, the EM density was fitted with two copies of hTERT and hTR, one on each lobe.

Further insights were gained from recent cryo-EM structures. The first cryo-EM structure of human telomerase with a telomeric DNA substrate determined at ∼8 Å resolution [57] revealed a similar bilobed architecture but a different composition from the previous negative stain structure [56] (Figure 2A,B). The telomerase holoenzyme was purified under gentle conditions using a two-step purification, first via hTR then via hTERT. Guided by activity assays and negative stain EM, the lysis/purification was optimized to enrich for compositionally homogeneous and highly active telomerase particles, followed by further optimization in cryo-EM sample preparation. Human telomerase structure was highly flexible, which was overcome by cryo-EM image processing. The resulting cryo-EM reconstructions at ∼8 Å resolution for both lobes revealed clear protein and RNA secondary structure features, allowing for the unambiguous fittings of homology models of 10 protein subunits and domains of hTR (Figure 2B). The subunits segregate into two lobes flexibly tethered by hTR. One lobe, named the catalytic core, is fitted with the crystal structure of the flour-beetle *Tribolium castaneum* TERT [59,60], *Tetrahymena* TEN domain [61], the medaka TRBD in complex with the CR4/5 domain of medaka TER [62] and the PK/t RNA model [63]. The other lobe, named the H/ACA lobe, is fitted with two copies of the archaeal H/ACA heterotetramer (dyskerin, NOP10, NHP2 and GAR1) [64], a TCAB1 homology model and the H/ACA domain of hTR. The structure confirmed that, like yeast and *Tetrahymena* telomerase [65,66], human telomerase also has monomeric hTERT/hTR composition.

**Figure 3.**
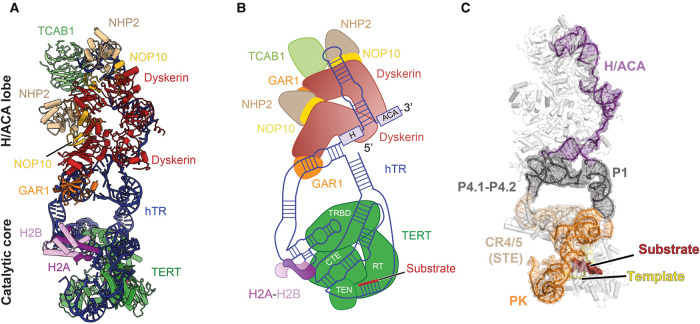
Structure of human telomerase holoenzyme bound to a telomeric DNA. (**A**) Human telomerase structure in cartoon representation with subunits coloured as indicated. (**B**) Cartoon schematic of the structure in (**A**). (**C**) hTR structure in human telomerase. Domains of hTR are coloured as shown in Figure 1B.

Taking advantage of recent method developments in cryo-EM [67–70], we further improved the cryo-EM reconstructions of human telomerase to 3.8 Å and 3.4 Å resolution for the catalytic core and the H/ACA lobe, respectively [71] (Figure 2C). This big leap in resolution yielded the first atomic model for the complex (Figure 3), and accounts for a vast majority of previous genetic and biochemical work. The resolution gain also allowed the identification of histone H2A–H2B dimer as novel telomerase subunits, which were previously in an unmodelled part of the 8 Å map. The structure illuminates an intricate network of protein–RNA and protein–protein interactions that hold the assembly of 12 protein subunits (hTERT, 2 copies of the H/ACA heterotetramers, TCAB1, H2A and H2B) and hTR together. The next sections of the review will discuss these interactions in more detail.

### The catalytic core

**Figure 4.**
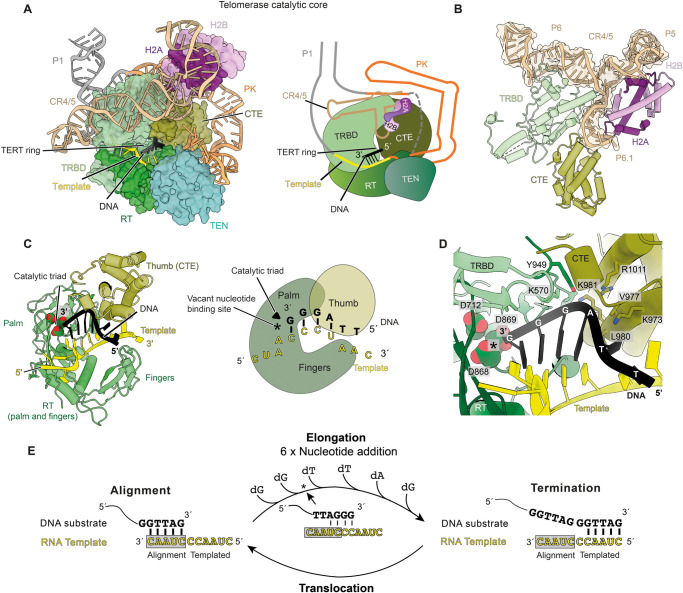
Human telomerase catalytic core. (**A**) An overview of human telomerase catalytic core. Left panel, the structure of the catalytic core. Proteins are shown in surface representation, and nucleic acids are shown in cartoon representation. Right panel, a cartoon schematic of telomerase catalytic core. Colour schemes are shown in Figure 1A,B are used for hTERT and hTR domains. (**B**) Interaction of the CR4/5 domain of hTR with the TRBD and CTE domains of hTERT and histone H2A–H2B. (**C**) Left panel, close-up view of the substrate-template duplex held in the active site by the palm, fingers and thumb polymerase sub-domains of hTERT. Right panel, a cartoon schematic of the left panel. The RT domain of hTERT harbours the palm and fingers sub-domains while CTE domain is also known as the thumb. The catalytic triad (D712, D868 and D869) are indicated. The vacant nucleotide-binding site is also indicated with an asterisk. (**D**) DNA substrate recognition by the TRBD, RT and CTE domains of hTERT. Specific side-chain interactions are also highlighted. The vacant nucleotide-binding site is indicated with an asterisk. (**E**) A simplified model of the repeat addition processivity catalytic cycle of human telomerase. The cycle consists of four main steps: alignment, elongation, termination and translocation. The structure shown in (**A**,**B**) captured the complex in an elongation state as indicated by the asterisk. The base-pairing lines drawn for the alignment and termination steps are hypothetical.

The catalytic core is made up of hTERT, histone H2A–H2B and the two catalytically essential domains of hTR, PK/t and CR4/5 (Figure 4A). Each of the four domains of hTERT (TEN, TRBD, RT and CTE) plays a unique role in telomerase function (Figures 1A and 4A). The TEN domain is crucial for repeat addition processivity and recruitment to telomeres [72–77]. The latter three domains form the TERT-ring [59] that accommodates the template-DNA duplex and is connected to the TEN domain via a flexible linker (Figure 1A). As its name suggests, TRBD provides high-affinity binding to hTR [78] (Figure 4A). hTERT has the right-hand shape with the fingers, palm and thumb sub-domains characteristic of most polymerases [79] (Figure 4C). The fingers and palm are contained within the RT domain of hTERT, which provides the polymerase catalytic site. hTERT polymerase thumb domain is also known as the CTE.

The PK/t and CR4/5 domains of hTR scaffold the domains of hTERT (Figure 3C and 4A). The TRBD and CTE of hTERT bear the majority of the interactions with hTR, except for the template region. Within the PK/t domain, the PK binds at the interface between the TRBD and CTE of TERT, and curves around the CTE domain to connect to the template (Figure 4A). The template is held by the palm and fingers within the RT domain (Figure 4C). The CR4/5, consisting of P5, P6 and P6.1 stems, adopts a Y-shaped conformation, facilitated by extensive interactions with the TRBD and CTE of TERT and histone H2A–H2B dimer (Figure 4B). The H2A–H2B dimer was not previously predicted and was only identified based on the cryo-EM density. In support of our identification, recent RNA proximity labelling studies show that hTR is enriched in histone H2B pulldown [80]. The P6.1 stem of the CR4/5 is highly conserved among vertebrates and essential for telomerase catalytic activity [29,81]. Remarkably, in our structure, P6.1 is cooperatively shaped by the TRBD, CTE and histone H2A–H2B (Figure 4B). Our structure suggests that the histone H2A–H2B dimer may play a role in assisting CR4/5 folding during telomerase assembly.

### Telomerase catalytic cycle

A proposed complex catalytic cycle allows telomerase to achieve its unique repeat addition processivity [17]. The catalytic cycle involves a series of alignment, elongation, termination and translocation steps and has been simplified in Figure 4E. To initiate telomeric repeat synthesis, the 3′ end of telomeric DNA base-pairs with the alignment region of the hTR template. This is followed by elongation, during which TERT sequentially adds six nucleotides to the 3′ end of the DNA substrate using the remaining 5′ half of the RNA template. The enzyme then terminates when it reaches the end of the RNA template and a full GGTTAG repeat has been synthesized. The product DNA must translocate to re-align with the alignment region for the another round of repeat synthesis.

In the cryo-EM structure of telomerase, a telomeric DNA primer terminating with the TTAGGG permutation was used due to its high-affinity interaction with telomerase [82]. This structure thus captures telomerase in the elongation phase of the catalytic cycle (Figure 4E) and reveals how this terminal TTAGGG repeat is accommodated within the active site of TERT (Figure 4C,D). The TRBD, RT and CTE domains are involved in DNA recognition (Figure 4D). The next template base is positioned to pair with an incoming deoxynucleotide triphosphate (dNTP). However, due to the absence of added dNTPs, this nucleotide-binding site is vacant within the determined structure (Figure 4C,D).

Until now, it was unclear how many base-pairs the DNA substrate and the RNA template can form in TERT active site at each stage of the telomerase catalytic cycle. During initiation, up to five base-pairs could form between the 3′ end of telomeric DNA and the alignment region of the template (Figure 4E). As the DNA substrate is elongated, the DNA substrate-template duplex could potentially lengthen. At the termination stage, the DNA product can theoretically form up to 11 base-pairs with the template based on sequence complementarity.

The TTAGGG terminal repeat could potentially form up to six base-pairs with the RNA template (Figure 4E). However, in the atomic model built into the cryo-EM map, only four base-pairs were observed (Figure 4C–E). Interestingly, interactions between the DNA and the thumb (CTE) domain of hTERT turn the TT nucleotides at the 5′ end of the DNA away from the RNA template instead of forming two additional base-pairs with the template (Figure 4C,D). This observation suggests that 3′ extension of the DNA substrate may occur concomitantly with 5′ duplex melting, and this partial duplex melting may be facilitated by the CTE domain. Consequently, during elongation, the DNA–RNA duplex may not lengthen, and the active site likely maintains a shorter DNA–RNA duplex than predicted (Figure 4E). Further work would be required to confirm this hypothesis.

### Telomerase H/ACA RNP

**Figure 5.**
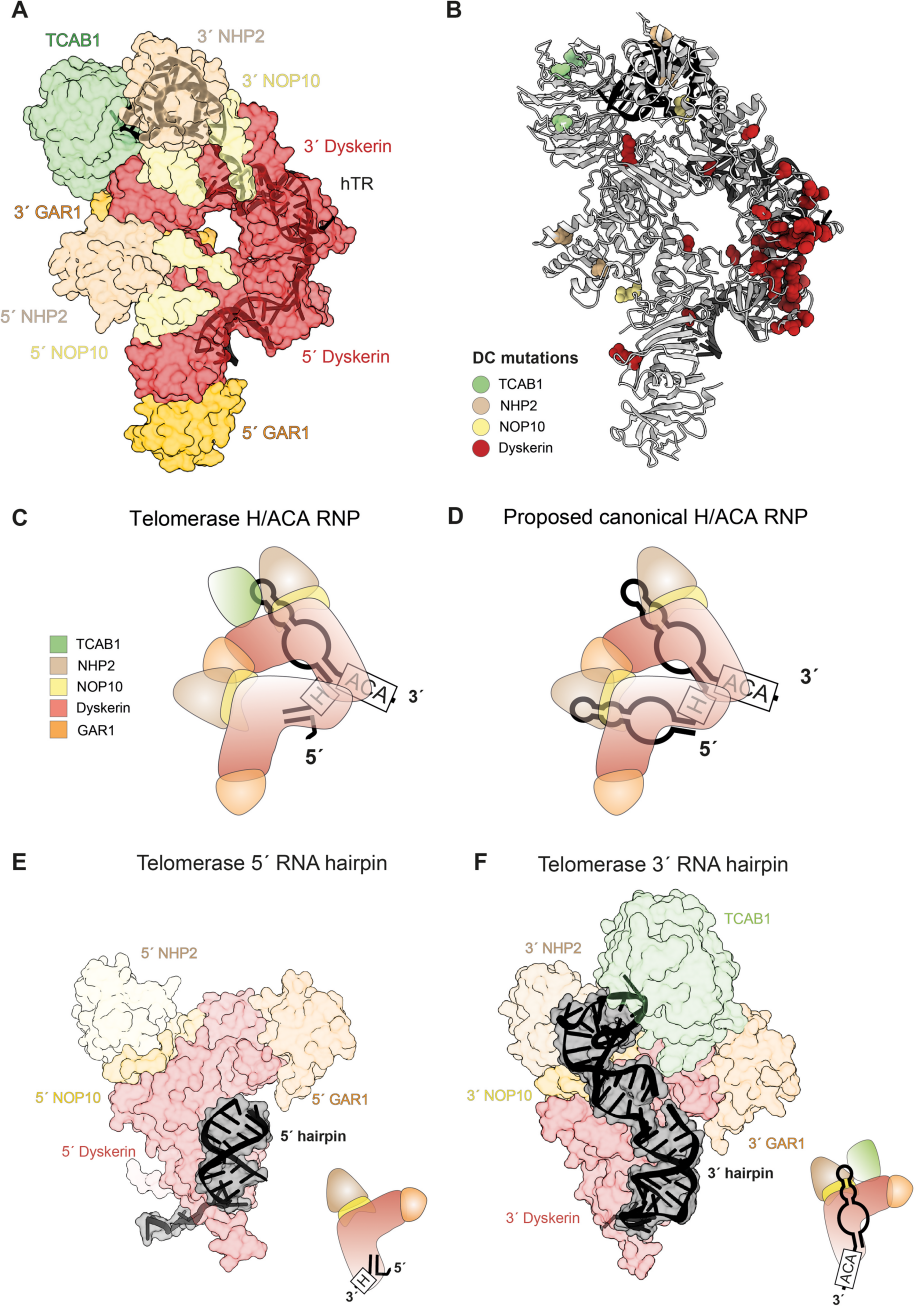
Telomerase H/ACA RNP. (**A**) Structure of human telomerase H/ACA RNP. Proteins are shown in surface representation and hTR is shown in cartoon representation. (**B**) Dyskeratosis congenita (DC) disease mutations mapped onto the human telomerase H/ACA RNP structure. Residues whose mutations are associated with DC disease are shown as spheres and coloured according to the associated subunits as indicated. (**C**,**D**) Cartoon schematics of telomerase H/ACA RNP and a proposed canonical H/ACA RNP, respectively. (**E**,**F**) Structures of the 5′ and 3′ H/ACA RNA hairpins of telomerase bound to their corresponding H/ACA heterotetramers, respectively. The cartoon schematics are also shown on the right corner of each panel. This illustrates the reduced protein–RNA interactions at the 5′ hairpin of telomerase H/ACA RNA.

Eukaryotic H/ACA RNAs, including vertebrate telomerase RNA, generally consist of two tandem RNA hairpins which assemble two copies of the H/ACA proteins, one on each hairpin (Figure 1C) [83]. This family of RNPs includes the small nucleolar RNPs (snoRNPs) and the small Cajal body RNPs (scaRNPs), which are responsible for pseudouridylation of ribosomal and spliceosomal RNAs, respectively [84]. The isomerization of uridine (U) nucleotide to pseudouridine (Ψ) is the most common post-translational modification of cellular RNAs and critical for various cellular processes such as ribosome and spliceosome biogenesis [85]. Crystal structures of single hairpin H/ACA RNPs from archaea and yeast provided great insights into how the H/ACA proteins assemble with one another and with the associated RNA hairpin containing only the ACA box [64,86]. It was not until the first 8 Å human telomerase structure was solved that the architecture of a full double-hairpin eukaryotic H/ACA RNP was revealed [57] (Figure 2B). The archaeal homologue was used for fitting into the density due to the lack of a human single hairpin H/ACA RNP structure. The recent 3.4 Å structure of the H/ACA RNP, as part of human telomerase, provided unprecedented molecular details regarding its assembly and the location of numerous human disease mutations (Figure 5A).

On its own, each H/ACA heterotetramer (dyskerin, NOP10, NHP2 and GAR1) forms a similar assembly as seen in previous structures [64] (Figure 5E,F). They are referred to as 5′ or 3′, depending on their association with the 5′ or 3′ RNA hairpin (Figure 5A). The binding of 3′ RNA hairpin of hTR to the corresponding H/ACA proteins resembles what has been observed previously with the single hairpin structure [64] (Figure 5F). RNA recognition is achieved by the 3′ dyskerin, NOP10 and NHP2 with additional stabilization from TCAB1 binding to the hairpin loop (Figure 5A,C,F). In contrast, the atypical 5′ RNA hairpin of telomerase contains only a binding site for dyskerin, not for NOP10 and NHP2, and thus deviates from the canonical binding mode (Figure 5A,C,E). Unexpectedly, the 5′ dyskerin, 5′ NOP10 and 5′ NHP2 extensively interact with the 3′ dyskerin and 3′ GAR1 (Figure 5A,C). The sub-optimal RNA–protein interaction at telomerase 5′ RNA hairpin (Figure 5E) is likely compensated for by this observed inter–tetramer interaction. This also brings the conserved H and ACA boxes into close proximity (Figure 5C), which had not been predicted previously.

The above observation has several important implications. Each of the two hairpins of the H/ACA RNA contributes differently to the H/ACA RNP assembly. Changes made to the 3′ hairpin, which reduce its protein binding affinity, were detrimental to the accumulation of both hTR and the canonical H/ACA snoRNAs [45]. On the other hand, many disruptions made to various regions of the 5′ hairpin did not affect telomerase activity and hTR accumulation [45]. Although the atypical 5′ hairpin is specific to hTR, deletions within the 5′ hairpin of snoRNAs designed to mimic the atypical 5′ hairpin of hTR were tolerated for snoRNA accumulation [45]. The cross–hairpin interactions observed in our structure account for this asymmetry of the 5′ and 3′ hairpin requirements for H/ACA RNA accumulation and are likely a general feature in all H/ACA RNPs (Figure 5C,D). This also suggests that during H/ACA RNP assembly, the 3′ hairpin assembles with the 3′ H/ACA tetramers first, which would subsequently allow the assembly of the 5′ hairpin counterpart.

The inter–tetramer interactions also explain why dyskeratosis congenita mutations found in the H/ACA RNP specifically result in telomere maintenance defects rather than ribosome and spliceosome biogenesis defects [87]. These disease mutations cluster at a hotspot at the interface between the two dyskerin molecules (Figure 5B). Given the lower protein affinity of the 5′ RNA hairpin of telomerase H/ACA, these mutations likely destabilize the interactions between the two H/ACA tetramers, resulting in the aberrant assembly of the H/ACA proteins on the 5′ hairpin. With a regular 5′ RNA hairpin, the effect of the mutations on the snoRNPs/scaRNPs would be less profound.

TCAB1 provides further protein–RNA affinity enhancement on the 3′ H/ACA hairpin of telomerase (Figure 5A,C,F). Here we observed and modelled the WD40 domain of TCAB1, which interacts with the CAB box of hTR and 3′ dyskerin and 3′ GAR1. The CAB box and TCAB1 are required for hTR localization to the Cajal bodies [46]. However, the exact roles of TCAB1 and Cajal bodies in telomerase regulation are under ongoing investigations. Interestingly, both the H/ACA RNP assembly pathway and Cajal bodies can be bypassed using a minimal telomerase RNP that contains hTERT and a minimal hTR construct lacking the whole H/ACA domain [88]. Additionally, TCAB1 knockout cells initially experience telomere shortening [46,88,89] but eventually maintain telomere length homeostasis; and these cells still have telomerase activity [88, 90]. These findings suggest that TCAB1 is not essential for the catalytic activity of telomerase, in agreement with it being distant from the catalytic core in our structure. In contrast, TCAB1 is suggested to play a role in the folding of the CR4/5; and its deletion results in misfolding of hTR and consequentially reduced telomerase activity [89]. Given the long distance between CR4/5 and TCAB1 observed in our structure, the mechanism underlying this observation has yet to be explained. Recent live-cell imaging studies demonstrate that in the absence of TCAB1, hTR and hTERT partition to different compartments in the nucleus, preventing telomerase assembly [90]. Thus, TCAB1 is proposed to be required for telomerase assembly. To fully appreciate the role of TCAB1, future studies would be necessary to understand how telomerase reaches its mature assembled state as observed in the structure.

## Prospects

Although we gained unprecedented mechanistic insights into human telomerase, when compared with the understanding of other RNPs gained through structural studies such as the ribosome [91] or spliceosome [92], telomerase structural studies are still in their infancy. Future structural work will continue to play fundamental roles in addressing many important aspects of telomere biology such as telomerase catalysis, regulation and biogenesis.

Many questions regarding how repeat addition processivity is achieved still remain to be answered: How does telomerase initiate on a telomeric DNA substrate? How many base-pairs are maintained in the active site at each stage of the catalytic cycle? What signals termination upon the addition of a full GGTTAG repeat? How is the product DNA substrate repositioned in the active site for another round of repeat synthesis? How do G-quadruplexes affect telomerase activity [93]? Although structures of *Tetrahymena* telomerase at different stages of the catalytic cycle have been recently captured [94], the human enzyme will require its own sets of snapshots for a thorough mechanistic understanding of its catalytic actions.

Telomerase recruitment to telomeres is mediated by the shelterin complex, which binds mammalian telomeric DNA specifically [15,77,95–97]. One of its components, TPP1 directly interacts with the TEN domain of TERT [76,77,95,96]. TPP1 also interacts with POT1, another shelterin component, which binds single-stranded telomeric DNA 3′ overhang [15]. Together, TPP1–POT1 greatly enhances telomerase processivity *in vitro* [98]. Future structural characterization of telomerase interaction with TPP1–POT1 and a telomeric DNA substrate will reveal the underlying mechanism of how TPP1–POT1 recruits and activates telomerase at the chromosome ends.

The association of histone H2A–H2B dimer with the catalytically essential CR4/5 RNA domain in human telomerase is unexpected [71]. Future investigation will help to clarify the roles of histone H2A–H2B in telomerase assembly and function and whether they have a more general role in RNA biology.

The biogenesis of cellular telomerase holoenzyme requires a complex pathway that is not fully understood [33, 99]. Provided that biochemical means to stall telomerase assembly could be developed, structural studies of telomerase at different stages of its assembly pathway could provide great mechanistic insight into the formation of the mature RNP. This could also address the outstanding question regarding the role of TCAB1 in telomerase assembly and biogenesis as discussed above.

## Conclusions

The low natural abundance, complexity and flexibility of human telomerase had made its structure determination an intractable problem for many years. The ‘Resolution Revolution’ in cryo-EM has allowed these hurdles to be overcome, and the telomerase field has entered the structural era. This first atomic view of human telomerase holds promise for drug design studies and provides a structural framework for future studies on human telomerase catalytic cycle, telomere recruitment and regulation.

## Perspectives

- Telomerase ribonucleoprotein resolves the end-replication problem to maintain genome stability. Telomerase dysfunction and misregulation are implicated in cancers and ageing. Therefore, it is crucial to understand how telomerase functions at a molecular level.
- The first detailed visualization of the human telomerase holoenzyme using cryo-EM accounts for numerous genetic, biochemical and cell biology data and provides mechanistic insight into telomerase assembly and function.
- Future structural studies combined with biochemical and biophysical methods and cell biology will be required to address further questions regarding the molecular mechanism and regulation of human telomerase.

## Open Access

Open access for this article was enabled by the participation of MRC Laboratory of Molecular Biology in an all-inclusive *Read & Publish* pilot with Portland Press and the Biochemical Society under a transformative agreement with JISC.

## Abbreviations

CTE: C-terminal extension
PK/t: pseudoknot–template
scaRNPs: small Cajal body RNPs
snoRNA: small nucleolar RNAs
snoRNPs: small nucleolar RNPs
STE: stem-terminus element
TEN: telomerase essential N-terminal
TERT: telomerase reverse transcriptase
TRBD: telomerase RNA binding domain
